# I Want More and Better Cells! – An Outreach Project about Stem Cells and Its Impact on the General Population

**DOI:** 10.1371/journal.pone.0133753

**Published:** 2015-07-29

**Authors:** Sara Varela Amaral, Teresa Forte, João Ramalho-Santos, M. Teresa Girão da Cruz

**Affiliations:** 1 Department of Life Sciences, University of Coimbra, Coimbra, Portugal; 2 CNC - center for Neuroscience and Cell Biology, University of Coimbra, Coimbra, Portugal; 3 Faculty of Psychology and Education Sciences, University of Coimbra, Coimbra, Portugal; Hospital Nacional de Parapléjicos - SESCAM, SPAIN

## Abstract

Although science and technology impact every aspect of modern societies, there is still an extensive gap between science and society, which impairs the full exercise of citizenship. In the particular case of biomedical research increased investment should be accompanied by parallel efforts in terms of public information and engagement. We have carried out a project involving the production and evaluation of educational contents focused on stem cells - illustrated newspaper chronicles, radio interviews, a comic book, and animated videos - and monitored their impact on the Portuguese population. The study of the outreach materials in a heterogeneous sample of the population suggests that they are valuable tools to disseminate scientific messages, and that this is especially true for the comic-book format. Furthermore, the data showed that clear and stimulating outreach materials, that are able to teach new concepts and to promote critical thinking, increase engagement in science at different levels, depending on the depth of the concepts involved. Additionally, these materials can influence political, social and personal attitudes toward science. These results, together with the importance attributed to scientific research in stem cells by the population sampled, validates the diffusion of such materials as a significant contribution towards an overall public understanding and engagement in contemporary science, and this strategy should thus be considered in future projects. Regardless, stringent quality control must be implemented in order to efficiently communicate accurate scientific developments, and the public stimulated in terms of finding additional sources of reliable information.

## Introduction

Science is present in almost every aspect of our daily life, affecting a wide range of public and personal issues. Scientific and technological advances have a significant impact on political stability, social well-being, economic growth and progress [1,2,3,4]. However, the interaction between science, politics and citizens is frequently insufficient, and sometimes non-existent. This lack of understanding and communication compromises the full exercise of citizenship in contemporary democratic societies [5,6].

Given the importance of lay audience awareness regarding scientific issues, and in order to bridge the communication gap between researchers and the general public, thus strengthening the links between science and society, improvements should be considered [7,6]. For example, the educational system needs to more closely follow scientific breakthroughs for the benefit of both students and teachers. Science awareness can also potentially facilitate informed and responsible decisions made by citizens, namely regarding health issues [1,8]. Furthermore, the understanding of what science is about, what it can (and cannot) do is crucial for its involvement in a democratic society [9,5]. On the other hand, governments must be motivated to engage scientists in problem-solving at this level, and, given that understanding and applying scientific developments in industry could increase competitiveness and productivity, decision makers must be aware of all relevant scientific issues, as well as of their possible implications. Therefore, improving the appreciation and trust in science and promoting public engagement is paramount towards guaranteeing sustained development [1,10].

The major developments in biomedical research in the 21^st^ century have led to novel concepts and ideas, that, in turned attracted interest from the general population [11]. In Portugal, health is one of the scientific areas that most interests the population– 72% of Portuguese society [12]. And in the last decades the increase of scientific research in Portugal has been notable, overcoming all expectations [13,14]. Consequently, general awareness concerning scientific issues grew as well. Other data indicates that the young Portuguese population tops the European list in terms of interest in science and technology– 86% comparing to an average of 67% for all European Union members [15]. It is thus a particularly receptive society in terms of efforts that may contribute to more knowledgeable engagements in science and health issues. Additionally, the Portuguese population believes that science and technology positively influences society (69% of the overall population, 86% of the young population between 15 and 24 years of age) [16]. However, the majority (67% of the Portuguese population comparing to 58% at the EU27 level) consider that suitable science information is lacking [16]. The majority (54%) also considers scientists in public research centers and universities as the most informed, and therefore the most adequate sources in terms of scientific information [16]. It is therefore clear that the communication and dissemination of scientific developments should be fostered by the scientific community, and that its role is vital [17,18,19,20]. Linking scientific and non-scientific communities must be part of the solution to increase scientific literacy, thus helping to fill the information gap. By supporting science outreach activities and by improving the communication skills of researchers and students we can promote not only the understanding of scientific issues by the public, but also increase the trust in science [21].

**Fig 1:**
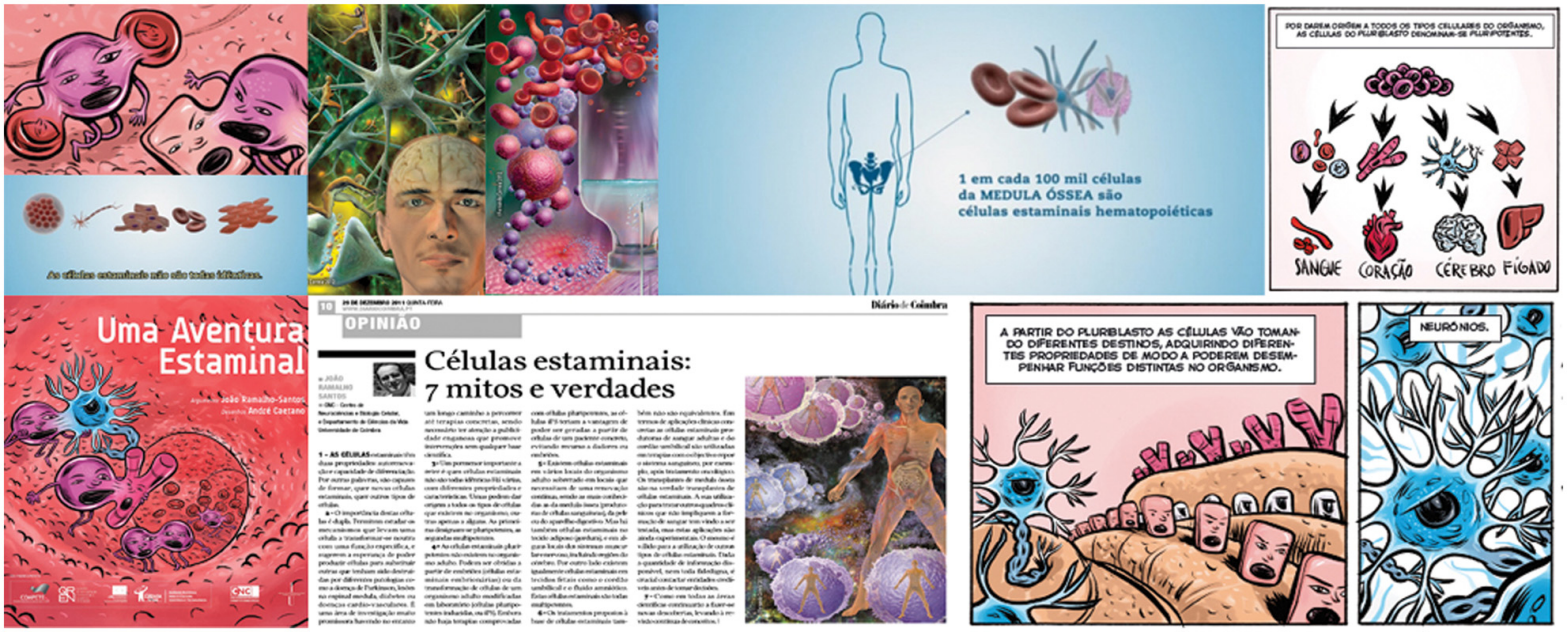
Outreach materials. Illustration of the materials produced in the project evaluated in this study: animated videos, newspaper illustrated chronicles, and comics. Reprinted from “Uma Aventura Estaminal” [28] and “Células Estaminais [29] with permission from André Caetano and Fernando Correia.

In the last decade, stem cell research has taken an important place in contemporary science and biomedicine, which makes this subject exceptionally suited for science communication. On that basis, researchers from the Center for Neuroscience and Cell Biology (University of Coimbra, Portugal) developed the science communication project “I want more and better cells! Stem cells: What are they? Where are they? What can they be used for?” for the production of outreach materials (Fig 1). Several aspects of stem cell biology and stem cell research were presented and discussed in these outreach materials namely: 1) origin and differentiation characteristics of different types of stem cells, 2) milestones in stem cell research, and 3) current and potential applications. Stem cells are considered particularly promising due to their unique characteristics of self-renewal and the ability to give rise to multiple specialized cell types [22,23,24]. Regarding stem cell differentiation characteristics, the fundamental concepts of pluripotency, multipotency and unipotency were presented. The establishment and development of this particular research field was discussed in term of key events, such as i) the establishment that cell differentiation is reversible [25]; ii) the development of the first human embryonic stem cell lines [24] and the beginning of extensive experimentation with these cells; and iii) the development of new methods to reprogram cells, i.e. to induce mature cells to reverse their developmental commitment [26]. Currently stem cells are a powerful tool to tackle unsolved problems, and can be potentially used to screen drugs and toxins, to test molecular targets of disease, as a model to study human development, and to develop new therapeutic approaches involving the replacement of damaged cells by new, stem-cell derived, counterparts. However, while the potential of stem cell-based technologies is immense [23], and thus attracts public attention, some aspects also raise ethical issues, somewhat mitigated by the lower reliance on human embryos [22,27]. However, the public must also be made aware that stem cells in no way represent a miraculous source of possible therapies for a number of ailments, but a challenging and demanding research area. In that regard the only uncontroversial and well-established clinical applications are bone marrow/cord blood transplants involving hematopoietic stem cells, notably to replenish blood formation following an oncological intervention.

Taking into account the developing interest in biomedical science and the recent boost in stem cell research, a multidisciplinary team involving specialists in stem cell research, science communication, scientific illustration, and journalism, undertook the challenge of developing outreach materials that could effectively communicate information about stem cells—comics, newspaper illustrated chronicles, radio interviews and animated videos, to be distributed nationwide. By working in an established field, such as the stem cell field, but using novel strategies and diversified tools, this team hoped to contribute towards a greater public engagement, thus promoting more informed decision making, and connecting science and the public in a permanent dialogue [30,9]. This type of outreach effort in this particular field has been growing worldwide with several initiatives such as science festivals (for example the Cambridge Science Festival) [31] and the production of science information materials—Comic books and Animated videos produced by EuroStemCell and Stem Cell Network [32,33,34,35]. This project is in line with these innovative science dissemination approaches, but it is important to highlight that, to our knowledge, this is the first outreach project about stem cells developed in Portugal and in the Portuguese language. The outreach materials were primarily produced to support the development of science literacy, a process that involves learning scientific issues and the nurturing of values and attitudes in society [36]. Although there are several views on this topic, we believe that the best way to improve scientific literacy doesn’t necessarily involve the production of more information about science, but rather more suitable communication approaches that might be better understood by target audiences. This must be carried out without compromising the accuracy of concepts that are being transmitted, but with a clear sense that not all concepts/materials are suitable for all audiences.

Along these lines, it is crucial to conduct evaluation studies that might shed light on how different educational materials are promoting knowledge, and fostering understanding, engagement, and possibly influencing attitudes towards scientific discoveries and applications. More specifically, and given that the materials for this particular project were not developed with a specific target group in mind, we wanted to explore possible differences in terms of how they were perceived by distinct audiences, thus identifying trends that might be useful in future initiatives of a similar nature.

## Methods

### Participants

For this evaluation study we chose a sample from the Portuguese population, in order to assess the impact of the outreach materials (described below), written and spoken in Portuguese. The choice of the sample size (n ≥ 200) was based on a minimum “n” recommended by the PLACES Impact Assessment Toolkit for our type of evaluation instrument (survey/questionnaire). The PLACES Impact Assessment Toolkit was created to measure the impact of initiatives and policies within the area of science communication and scientific culture [37]. Two hundred and six subjects participated in this study (Table 1), 45.1% men (n = 93), and 54.9% women (n = 113). Participants were between 14 and 85 years of age with a mean age of 36.39-years (median = 25-years old). The sample included individuals randomly chosen from distinct groups established according to their age, education, and economic sector of activity: 1) 25.3% were high-school students (n = 52): 11.7% of those from science-based courses (n = 24), and 13.6% from humanities and arts courses (n = 28); 2) 27.7% were University students (n = 57): 13.69% from biomedicine-related academic majors (n = 28) and 14.1% from non-biomedicine-related academic majors (n = 29); 3) 23.7% belonged to the active population (composed by individuals from the three sectors of activity); and 4) 23.3% are retirees (n = 48). In order to obtain a broader sample with individuals with different ages and expertise, we chose to have a convenience sample. However, in each group the individuals were randomly selected according to their availability at the partner organizations. The entire sample was collected at local and regional institutions and organizations: a high school, university students associations, senior universities, a factory, and a community center. Although we acknowledge the limitation inherent to the use of a convenience sample that does not allow the generalization and inference regarding the entire Portuguese population, this type of sample was chosen in order to include a broader range of subjects with heterogeneous age and expertise, avoiding the risk of over representation of one or more groups and aiming at the construction of a more complete image of the Portuguese population.

**Table 1:** Socio-demographic characterization of the population sample.

	Frequency	%
**Gender**		
Male	93	45.1
Female	113	54.9
**Groups**		
***High-school students***		
Sciences	24	11.7
Humanities and Arts	28	13.6
Total	52	25.3
***University students***		
Biomedicine-related courses	28	13.6
Non-biomedicine-related courses	29	14.1
Total	57	27.7
*** Active population***	49	23.7
***Retired population***	48	23.3
**Total**	**206**	**100**

### Materials and procedures

The outreach materials evaluated in this study were developed by researchers from the Center for Neuroscience and Cell Biology (University of Coimbra, Portugal). The project that gave rise to the production of these contents intended to contribute towards a more knowledgeable society, that should be able to express informed opinions and make conscious decisions about stem cell research and its applications. Additionally, one of the goals of the project was to point out to non-scientific audiences that scientific research in this field is rapidly growing worldwide, developing novel biomedical research tools and potential therapies. In order to accomplish these goals, and as discussed previously, different outreach materials about stem cells and their applications were produced, namely a comic book, newspaper illustrated chronicles, radio interviews and animated videos.

The construction of the evaluation scheme was based in the Logic Model of Evaluation [38] and inputs, activities, outputs and outcomes were defined (S1 File) in terms of the main strategic impact pursued: the increase of scientific literacy on stem cells in the Portuguese society. For this purpose we conducted a quantitative evaluation using surveys, designed considering four main dimensions, already used in other evaluation studies of science communication initiatives [14]: 1) acquisition of knowledge, 2) understanding, 3) engagement, and 4) attitude. After contacting the partner institutions that collaborated in the sample selection/collection, we organized two sessions for each of the groups described in Table 1. During the first session participants were asked to fill out a first questionnaire (S2 File) comprising two sections: i) in the first section participants were introduced to the evaluation study and were asked to write three words or expressions that they commonly associated with stem cells; ii) the second section included 7 items of true/false (dichotomous questions), and 4 items of multiple-choice questions, all related to specific knowledge about stem cells (example: Stem cells are all equal: true or false; Physical and mental exercise leads to the formation of new neurons because in the brain there are: neural stem cells, hematopoietic stem cells, neurons or embryonic stem cells?). Subsequently, the researchers showed two of the four outreach contents (radio interviews and animated videos), and offered the book (that includes both the comics and the illustrated newspaper chronicles) to the participants to read until the next session. The sessions lasted 45–60 minutes. Two to three weeks after the first session the participants were asked to fill out a second questionnaire (S2 File), with a section matching the first questionnaire (for the assessment of scientific knowledge) in addition to three more sections designed to assess: i) the understanding of scientific topics (5 items), ii) the engagement with science (3 items), and iii) the attitude toward scientific issues (4 items). A five point Lickert Scale—totally disagree to totally agree, or very negative to very positive—was used to rank the responses for each of the second survey questions, except for one dichotomous question and two multiple choice questions.

### Statistical analysis

Four main studies were carried out with data collected from the same sample. For the first study, aimed at exploring knowledge acquisition, a mixed design with assessment time was used (a session before and a session after the contact with the materials), as a “within-subjects” variable, and population groups as the between-subjects factor (Mixed ANOVA). With the second study we intended to explore the effectiveness of the outreach materials in promoting understanding, engagement and changes in attitude in the different groups. In order to do so two MANOVAs (multivariate analysis of variance) were performed with the three dependent variables of understanding, engagement and attitude, and population groups as the “between subjects” factor. Descriptive statistics was performed to measure the central tendency (mean values of the response ratings for the variables analyzed), and to measure the variability of the response rating (standard deviation). In the third study we explored how the variables were associated through a correlation study. The fourth study explored general participant perceptions concerning the materials and research developed in the stem cells field. All statistical analyses were performed using the SPSS 20 statistical package (IBM).

### Ethics statement

The authors of the manuscript “I want more and better cells!—An outreach project about stem cells and its impact in the general population”, Sara Varela Amaral, Teresa Forte, João Ramalho-Santos, and M. Teresa Girão da Cruz, hereby state that, given the specific nature of the study, no approval was sought from an institutional review board or ethics committee. All participants (or parents, via caretakers, in the case of minors) verbally consented to participate. All participants were volunteers. This study was an anonymous questionnaire-based research project in which participants were asked to assess scientific educational materials geared at the general population, and presented in distinct platforms, in order to determine what was learned following this exposure. Data were analyzed anonymously. The study was carried out in the context of Science in the Classroom and Science & Society initiatives. No other contact was made, and no personal information of any sort was kept on file.

## Results

### Study I—Contribution of the outreach materials for knowledge acquisition

In order to explore if the outreach materials contributed towards knowledge acquisition in our sample, the eleven items corresponding to knowledge on stem cell topics of both questionnaires were computed into two variables named *Previous Knowledge* (with the proportion of correct responses for the first session questionnaire) and *Posterior Knowledge* (with the proportion of correct responses for the second session questionnaire, after contact with the outreach materials). The coefficients of internal consistency are moderate/acceptable for both variables, with a Cronbach’s alpha of 0.586 and 0.683, respectively, which suggests that the eleven items appear to measure the same general variable of knowledge.

To explore the differences between the two evaluation moments a repeated measures ANOVA was performed. The results showed that the average scores between the first time point (*M* = 0.439, *SD* = 0.014) and second time point (*M* = 0.794, *SD* = 0.012) were statistically significantly, *F* (3, 197) = 621.574, *p* < 0.001, *η^2^*_*p*_ = 0.759. Post hoc tests using the Bonferroni correction revealed that after contact with the outreach materials there was a statistically significant knowledge acquisition (*p >* 0.001).

**Fig 2:**
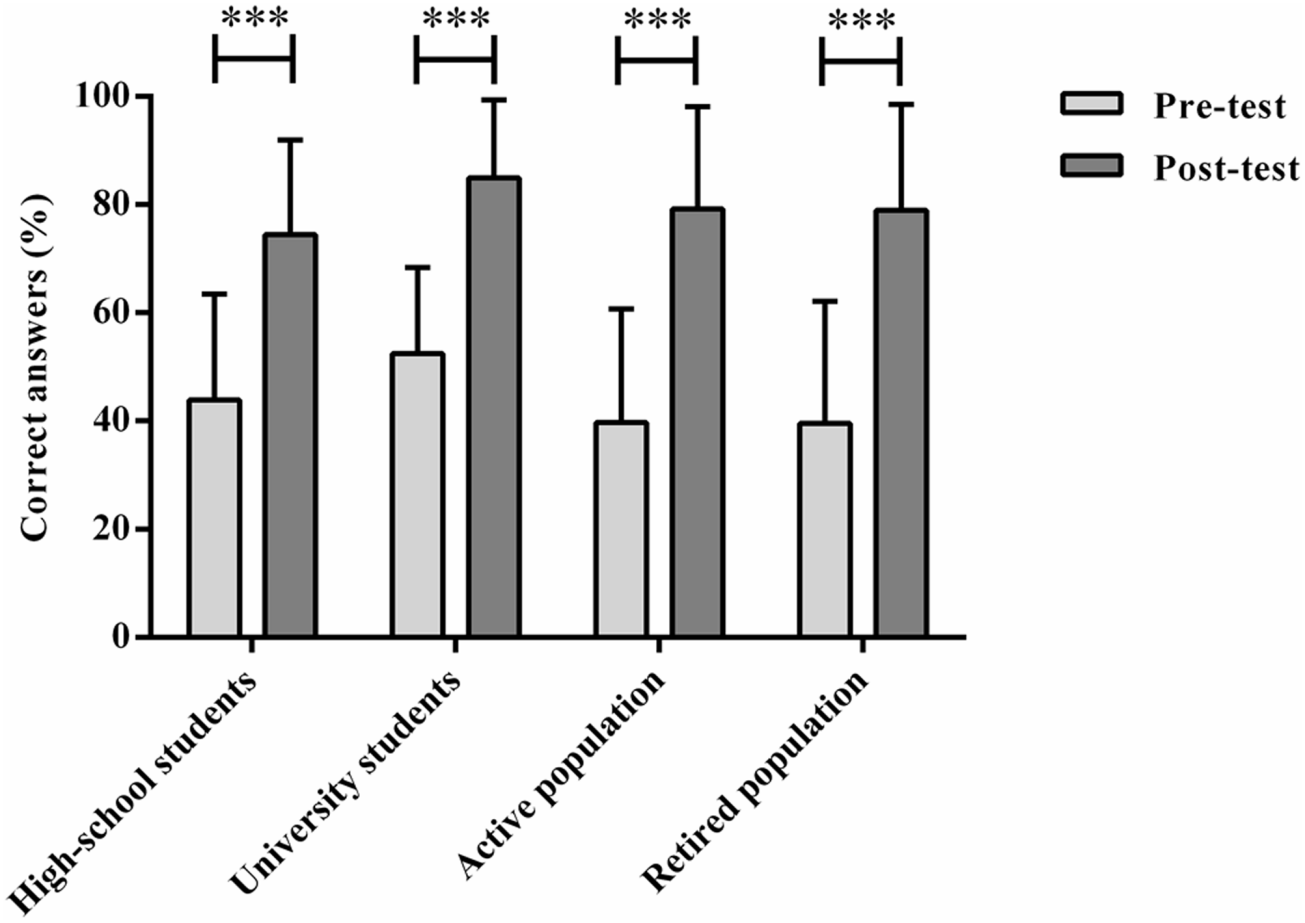
Percentage of correct answers related to the knowledge questions. Percentage of correct answers of the items related to the previous and posterior knowledge in the four groups analyzed. Repeated-measures ANOVAs were performed to explore the differences between the two evaluation moments, "***" *p* < 0.001.

Significant differences were also found between the mean values of each group from the first to the second evaluation time point. Separate repeated-measures ANOVAs were performed, showing that the knowledge increase was statistically significant for all the groups (Fig 2): high-school students, *F*(1, 51) = 147.19, *p* < 0.001, *η^2^*_*p*_ = 0.743; university students, *F*(1, 51) = 169.07, *p* < 0.001, *η^2^*_*p*_ = 0.768; active population, *F*(1, 48) = 131.84, *p* < 0.001, *η^2^*_*p*_ = 0.733; retired population, *F*(1, 47) = 185.26, *p* < 0.001, *η^2^*_*p*_ = 0.798. These outcomes strongly suggest that the outreach materials were suitable for the transmission of knowledge on stem cells regardless of the age, expertise and professional background of the subjects involved.

In order to explore the possible role of the subjects academic field and expertise in more detail, a mixed ANOVA was conducted for the groups of high-school and university students, divided into Sciences (n = 24) and Humanities and Arts students (n = 28) and Biomedicine- related courses (n = 28), and Non-Biomedicine-related courses students (n = 29), respectively. All the groups showed a significant improvement in knowledge between the first time point and the second time point, *F* (1, 100) = 323.447, *p* < 0.01 *η^2^*_*p*_ = 0.764. Furthermore, there were no significant differences between groups, *F* (1, 100) = < 1, *p* = 0.106 *η^2^*_*p*_ = 0.021, which suggests that the proximity of the subjects with these themes in their academic fields did not have an effect on knowledge acquisition in this particular case. This result reinforces the suitability of the materials produced in promoting knowledge across subject groups with different backgrounds.

**Fig 3:**
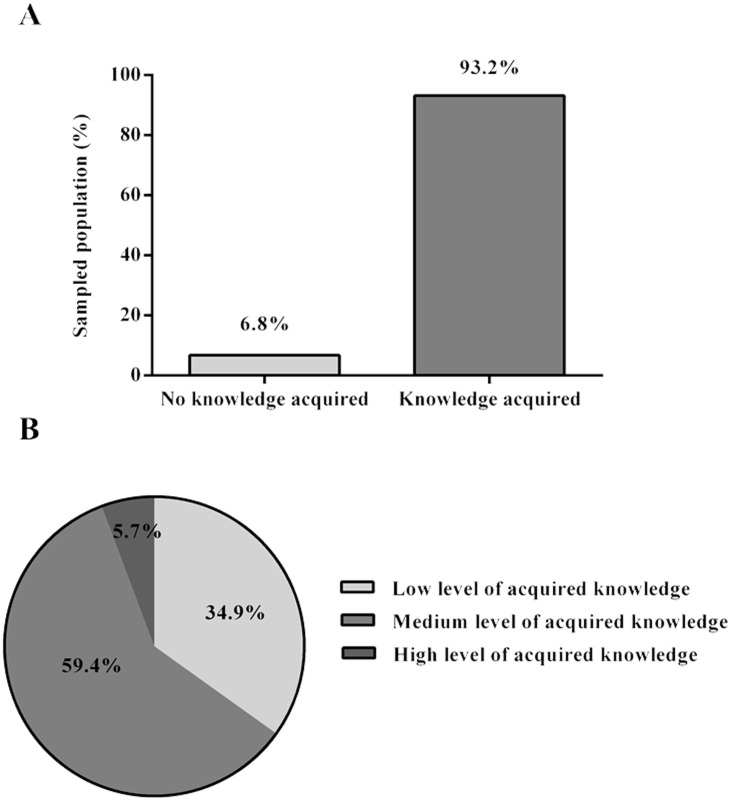
Knowledge Score. (A) Percentage of the population that acquired and that did not acquire knowledge after contact with the materials—general knowledge score. Knowledge acquisition was measured using the final knowledge score (an index that uses a scale between zero and one—zero representing no knowledge acquisition and one representing maximum knowledge acquisition). (B) Levels of acquired knowledge (%): low level of acquired knowledge, medium level of acquired knowledge, and high level of acquired knowledge; these levels were defined according to the final knowledge score of the total population. The graphs represent the data for all groups of the population sample.

A *Knowledge Score* was computed via the difference between *Posterior Knowledge* and *Previous Knowledge* comprising two categories: *knowledge acquired* and *no* k*nowledge acquired* (Fig 3). The results suggest that 93.2% of the population acquired some knowledge, and that 6.8% didn’t show improvement from the first to the second session (Fig 3A). Within the category *knowledge acquired*, in a scale ranging from zero to one, 34.9% of the total population presented a low level of acquired knowledge (0 to 0.33), 59.4% presented a medium level of acquired knowledge (0.33 to 0.66), and 5.7% had a high level of acquired knowledge (0.66 to 1) (Fig 3B).

### Study II—Contribution of the outreach materials for the Understanding, Engagement and Attitude towards stem cell research

The 8 items of the questionnaire defined to analyze the variables Understanding, Engagement and Attitude were submitted to a confirmatory factor analysis with a varimax rotation. The sample was suitable for a factor analysis according to the Kaiser-Meyer-Olkin Measure of Sampling Adequacy (KMO = 0.837) and the supposition test of sphericity by Bartlett`s test which is rejected at a level of statistical significance of *p* < 0.005 for Approx. Chi Square = 591.077.

From this analysis three factors were extracted (S3 File), with 72.1% of common variance between them. The first dimension includes the three items related to the Understanding of the scientific concepts: clarity, interest, and critical thinking; the second dimension includes the three items related to the Engagement variable: learning new concepts, curiosity, and wish to learn more; and the third dimension includes the two items concerning the Attitude variable: attitude (personal/socio-political decisions), and attitude (personal decisions). A Cronbach’s alpha of 0.76, 0.74 and 0.69 was found, respectively, for each of these dimensions.

As shown in Table 2 all the mean values for each variable in all groups were perceived to be above the midpoint of the scale (p > 0.001), 2.5 in a scale ranging from 1 (totally disagree) to 5 (totally agree). A one-sample *t-test* confirmed that the means are significantly above the mid-point of the scale. These results suggest that there was a high level of understanding, engagement and attitude in each group analyzed.

**Table 2:** Mean (*M*)^a^, standard deviation (*SD*), and independent sample t-test of the response ratings for all the groups in the population sample.

**High-school students**							**Active population**		
	Sciences		Humanities and Arts						
	*M*	*SD*	*M*	*SD*	*F*	*p*		*M*	*SD*
Understanding	4.03	0.56	3.40	0.78	3.26	0.02	Understanding	4.19	0.52
Engagement	4.01	0.51	3.38	0.59	4.13	0.00	Engagement	4.21	0.37
Attitude	4.04	0.33	3.54	0.68	3.33	0.02	Attitude	4.05	0.53
**University students**							**Retired population**		
	Biomedicine-related courses		Non-biomedicine-related courses						
	*M*	*SD*	*M*	*SD*	*F*	*p*		*M*	*SD*
Understanding	4.17	0.39	4.15	0.49	0.146	0.88	Understanding	4.04	0.59
Engagement	3.96	0.54	4.14	0.32	-1.49	0.14	Engagement	4.08	0.56
Attitude	3.96	0.58	4.19	0.36	-1.77	0.82	Attitude	4.10	0.65

Mean (*M*)^a^, standard deviation (*SD*) and independent sample t-test of the response ratings for High-school students (n = 52), University students (n = 57), Active population (n = 49), and Retired population (n = 48).

^a^All the means differ significantly from the mid-point of the scale (2.5).

A MANOVA was used to compare the means of the dependent variables of understanding, engagement and attitude between the four groups analyzed. We found significant differences in all the dependent variables, F (3, 193) = 5091.006, *p* < 0.01, *η^2^*_*p*_ = 0.988. More specifically, there were differences between groups for the variables Understanding, *F* (3, 195) = 8.317, *p* < 0.01, *η^2^*_*p*_ = 0.113; Engagement, *F* (3, 195) = 9.824, *p* < 0.01, *η^2^*_*p*_ = 0.131; and Attitude, *F* (3, 195) = 9.824, *p* = 0.012, *η^2^*_*p*_ = 0.055.

Moreover, post-hoc tests using the Bonferroni correction revealed that the differences between groups were mainly due to the means obtained for the high-school students.

**Fig 4:**
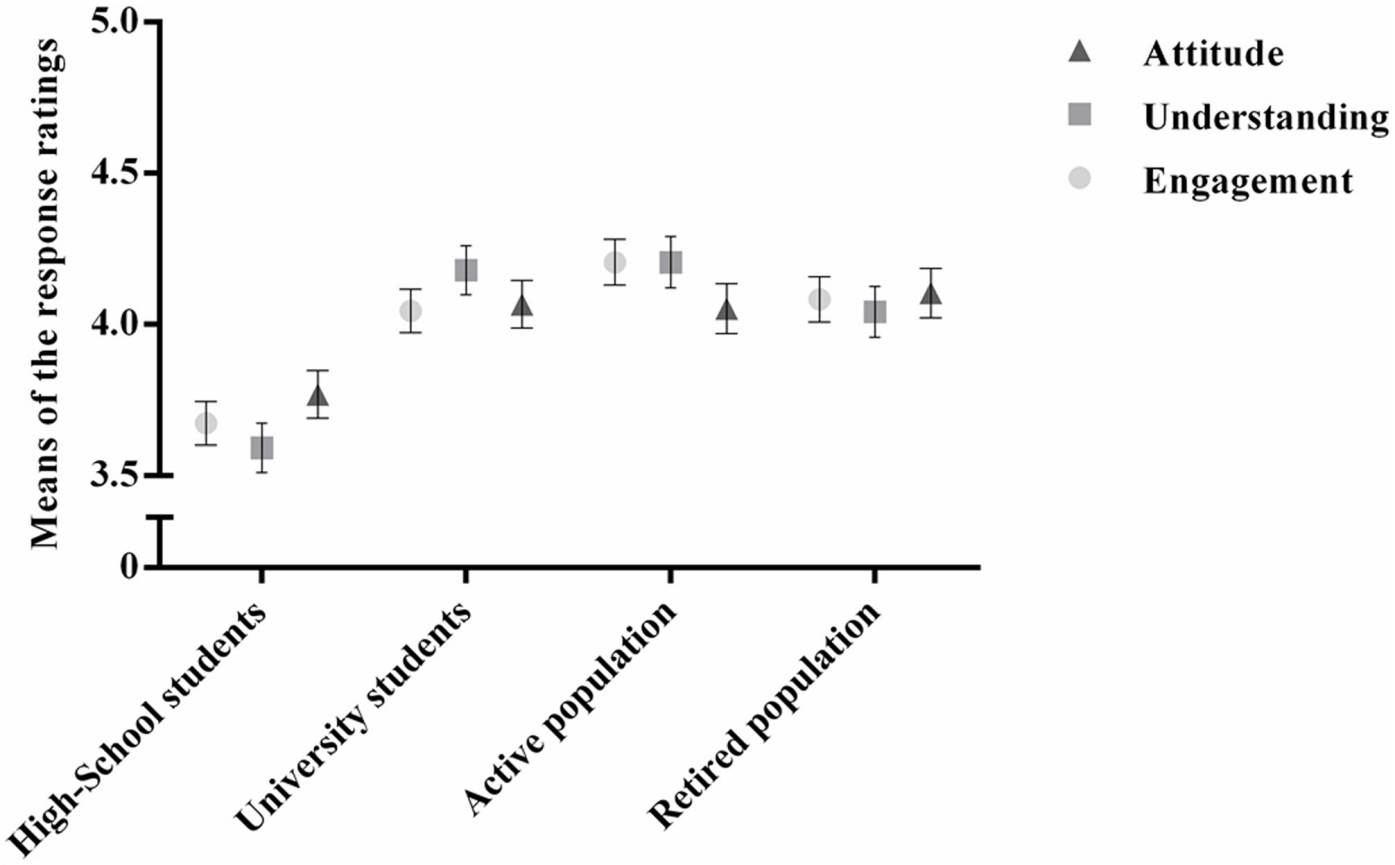
Understanding, Engagement and Attitude towards stem cells. Mean and standard deviation of the variables Understanding, Engagement and Attitude in the four groups analyzed.

In order to better understand these differences another MANOVA was performed after splitting the group of high-school students into two groups according to their field of study, Sciences (n = 24) and Arts and Humanities (n = 28). Similarly, we split the group of University students into Biomedicine-related courses (n = 28) and Non-Biomedicine-related courses (n = 29). These groups will be henceforth heuristically referred to as simply Scientific and Non-Scientific groups, respectively, so as to be equivalent to the division of the high-school students. Post-hoc tests using the Bonferroni correction revealed that the significant differences found for the groups in all the dependent variables are due to the group of arts and humanities students, which had lower means than the mean for the remaining groups (*p* < 0.05) (Fig 4). Despite the lower level of understanding and engagement with the materials, there was still an acquisition of knowledge in this group (Study I).

### Study III—Correlations between all variables (Knowledge, Understanding, Engagement, Attitude) towards stem cell research and applications

Correlation studies (Pearson´s correlation) were conducted in order to evaluate the strength (*r*) and significance (*p* < 0.05) of the associations between all the variables analyzed. In the present study, the interpretation of correlation strength was performed according to Pestana and Gageiro [39]. As shown on Table 3, the variables of Understanding, Engagement, Attitude, and Knowledge were all significantly and positively correlated when the population sample was analyzed as a whole, even if each of the different population groups showed different correlations (S4 File). It is noteworthy to highlight the strong correlation between Understanding and Engagement [*r* (206) = 0.62, *p* < 0.01], and between Engagement and Attitude [*r* (204) = 0.52, *p* < 0.01]. The Knowledge score shows a higher level of association with Understanding [*r* (206) = 0.22, *p* < 0.01].

**Table 3:** Pearson’s Correlations among all variables analyzed for the total population.

	Understanding	Engagement	Attitude	Knowledge score
Understanding	1			
Engagement	0.615**	1		
Attitude	0.484**	0.516**	1	
Knowledge score	0.219**	0.142*	0.156*	1

** Correlations are significant at the 0.01 level (2-tailed)

* Correlation is significant at the 0.05 level (2-tailed)

### Study IV—Participant perceptions

**Fig 5:**
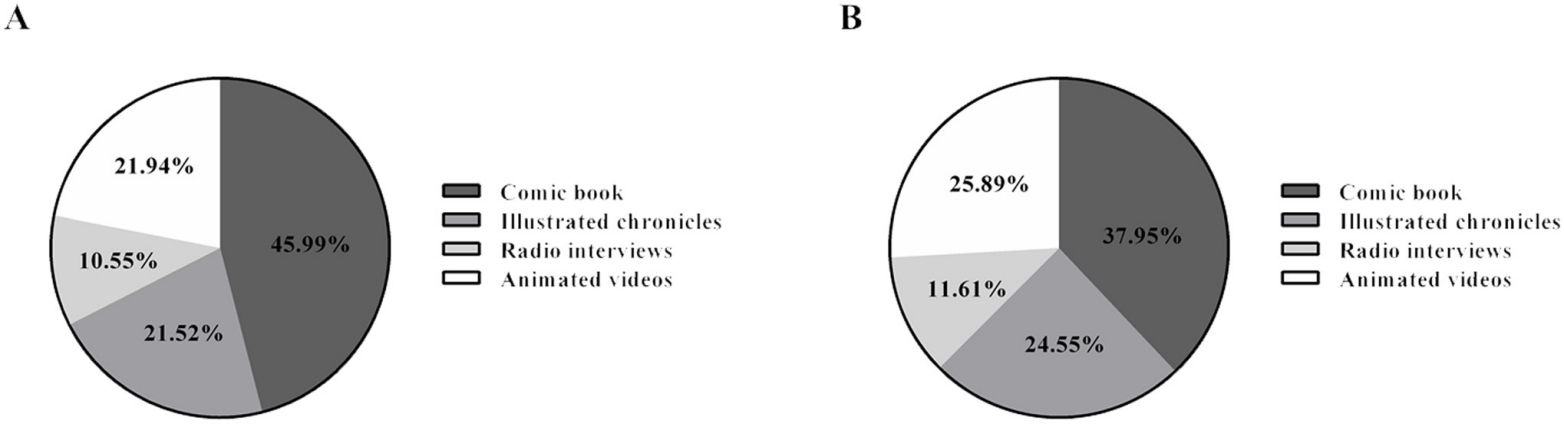
Preferences of the population towards the different outreach materials. Preferences of the population in terms of the different materials: comics, newspaper illustrated chronicles, radio interviews and animated videos. (A) % of total population that considered each of the outreach materials the one that contributed most towards the understanding of scientific issues; (B) % of total population that considered each of the outreach materials the one that contributed most towards an engagement with science.

All participants were asked to separately evaluate the different outreach materials about stem cells and their applications—the comic book, newspaper illustrated chronicles, radio interviews and animated videos—regarding their contribution towards their understanding and engagement. As shown on Fig 5A, the comic book was considered to be the outreach material that contributed most for a better understanding of the stem cells subject by 46.0% of the total population, followed by the illustrated chronicles (21.5%). The comic book was also considered the outreach material that had more impact on engagement regarding by 38.0% of the total population (Fig 5B).

When asked if they had any previous knowledge of the stem cell research, 70.4% (n = 145) of the participants answered positively, and 29.6% (n = 61) answered negatively. Among the participants that had some knowledge about this topic (n = 145), on a scale of 1 (*very negative*) to 5 (*very positive*), 0% had a very negative perception of the subject, 4.8% had a negative perception, 28.8% had neither a negative nor a positive perception, 53.4% had a positive perception, and 13.0% had a very positive perception. When the participants with previous knowledge were asked if the materials contributed to clarify and improve what they knew about the topic, 0.7% disagreed, 4.2% neither disagreed nor agreed, 60.4% agreed, and 34.7% totally agreed. When the participants that had “no knowledge” about this topic (n = 61), were asked to evaluate their perception after having been in touch with all the outreach materials, on a scale of 1 (*very negative*) to 5 (*very positive*), 8.5% had a negative perception, 28.8% had neither a positive nor negative perception, 49.2% had a positive perception, and 13.6% had a very positive perception. When asked about the importance of research in this field, on a scale of 1 (unimportant) to 5 (very important), 69.9% of the total population considered it as very important, 23.3% as important, 6.3% as relatively important, and 0.5% as not very important.

## Discussion

The science communication project “I want more and better cells!” developed by researchers from the Center for Neuroscience and Cell Biology at the University of Coimbra, Portugal, resulted in the production and dissemination of outreach materials on the subject of stem cells and stem cell research. This project appears to follow the one-way understanding and acquisition of knowledge model, placing it on the Public Understanding of Science movement [1], one of the established science communication approaches. However, taken together, the data from the quantitative evaluation suggests that the outreach materials can have an impact on Portuguese society, in terms, not only of learning and understanding of scientific topics, but also as it pertains to engagement and attitude towards science, indicating that the project can potentially contribute to the dialog model of science communication, also known as Public Engagement in Science [40,4]. In particular, the comic book was identified as the outreach material that provided the most input towards increasing the understanding of scientific issues related to stem cells, and to promote an overall engagement in related scientific matters. Perhaps not surprisingly, the use of this less common and innovative approach proved to be more suitable for communication purposes, although the more conventional communication materials (interviews, newspapers chronicles and videos) were also very positively evaluated. The specific efficiency of the comic book should therefore be taken into account in further initiatives of this kind. Regardless, care should be taken to ensure permanent accuracy and appropriate depth, and certain metaphorical strategies (such as the use of anthropomorphic elements) have to be well thought out in order to avoid the transmission of erroneous information, and may not be appropriate for all researchers or situations.

The style and the quality of the outreach materials contributed towards reaching the project goals, while matching societal needs. The study on knowledge acquisition showed that the great majority of participants surveyed acquired some level of knowledge, and the majority of those scored for medium or high levels of acquired knowledge, again suggesting the usefulness of the outreach materials to convey scientific concepts, and provide teaching tools. This overall analysis is reinforced by the effectiveness of the outreach materials in contributing towards understanding, engagement and attitude, in terms of this particular research topic. Furthermore, all the variables were strongly associated, suggesting that they have a tendency to change together. This should be taken into account in the development of effective scientific outreach materials in further initiatives.

Some differences between the different groups of the sampled population are worth highlighting. The high-school students typically scored significantly high for all the Understanding, Engagement and Attitude variables. Although Science students had higher scores than the Arts and Humanities students—probably related to the fact that these topics are usually addressed on the school curriculum of the former—all the results are significant, and indicate the receptiveness of these scientific issues, in accordance with previous findings regarding the young Portuguese population [16]. Taken together, the University students (both Biomedicine-related and Non-biomedicine-related academic majors) scored significantly high for all the variables analyzed *per se*, with no significant differences between the two groups. For the active population, composed by individuals from the three activity sector groups, the high mean scores for all the Understanding, Engagement and Attitude variables are especially noteworthy, suggesting that the outreach materials are suitable for a wide range of individuals, with different backgrounds, occupations and expertise. Regardless of the singularity of the retired population group, almost exclusively composed of individuals from senior universities who express high levels of interest and curiosity on a wide range of topics, the highest and strongest correlations found between the variables Understanding, Engagement and Attitude are still rather remarkable. We can most likely correlate this with the aptitude of older adults to continuously learn and engage [41,42,43], and their proneness to be aware of health issues and the potential impact of cutting-edge biomedical research on their lives.

Although stem cell research and stem cell use have been involved in public controversy, and the majority of the Portuguese population feels that it is not sufficiently informed about scientific topics [12,16], from our data we can infer that a broad range of individuals with different age and educational background considers stem cell research to be important, and already has some sort of understanding, as well as a positive perception of the field. This could be related to the fact that stem cells are currently a “hot topic”, often mentioned in the media, a subject of public debate, and a prospective hope for the treatment of several diseases, although possible applications beyond the established hematopoietic stem cell transplantation paradigm remain unlikely in the foreseeable future. However, the majority of our sampled population still expressed that the outreach materials provided were helpful in clarifying puzzling concepts and misunderstandings, and acquired some level of knowledge. Taken as a whole, we find these results particularly encouraging and, together with the great interest of Portuguese population in science and technology [12,16], the momentum should be used to bring together science and society and to explore innovative projects in Portugal, such as fundraising for biomedical research [44], science/society collaborative projects, participative projects, functional interactions between public, policy makers and scientists, and public engagement events, among others.

Further studies on a larger population sample could be of value to explore in greater depth the associations between the different variables analyzed, in order to better understand the magnitude of the impact of these materials in the population. Furthermore, the accurate communication of scientific developments must always be a paramount concern, and the public stimulated in terms of finding additional sources of reliable information to complement what can reasonably be transmitted in these sorts of activities. Regardless, the appreciation and high opinion of the Portuguese society regarding scientific research is an indicator that we are on the right path towards constructing a truly scientifically literate citizenship [7] and a more knowledgeable and engaged society [5].

## Supporting Information

S1 File Evaluation scheme designed for the project, based in the Logic Model of Evaluation. (PDF)

S2 File Questionnaires for the public sample (pre-test and post-test). (PDF)

S3 File Rotated factor matrix obtained by an exploratory factor analysis with varimax rotation for 8 items. The table contains the rotated factor loadings, which represent how the items are weighted for each requested factor (F1, F2, F3), and also the correlation between the items and the factor. The mean (*M*) and standard deviation (*SD*) for each item are also shown.
(PDF)

S4 File Pearson´s Correlations among all variables analyzed for the high-school students, university students, active population, and retired population. (PDF)
