# Supplementary material for: I Want More and Better Cells! – An Outreach Project about Stem Cells and Its Impact on the General Population
Source: PLoS One. 2015 Jul 29;10(7):e0133753. doi: 10.1371/journal.pone.0133753 (PMC4519251; doi:10.1371/journal.pone.0133753)
Supplement: S1 File — (PDF) [file pone.0133753.s001.pdf]

Evaluation scheme designed for the project, based in the Logic Model of Evaluation.

Inputs	Activities	Outputs	Outcomes	Strategic impact
Funding<br><br>Researchers with great expertise in the stem cells field<br><br>Science communication strategies	Production of outreach materials about stem cells and their applications:<br>Comic book<br>Newspaper illustrated chronicles<br>Podcasts<br>Animated videos	Dissemination of the outreach materials throughout the Portuguese population<br><br>Quantitative evaluation to a sample from Portuguese population (n=206)	Knowledge<br><br>Understanding<br><br>Engagement<br><br>Attitude	Improvement of scientific literacy of the Portuguese population in the stem cells field
