# Supplementary material for: I Want More and Better Cells! – An Outreach Project about Stem Cells and Its Impact on the General Population
Source: PLoS One. 2015 Jul 29;10(7):e0133753. doi: 10.1371/journal.pone.0133753 (PMC4519251; doi:10.1371/journal.pone.0133753)
Supplement: S2 File — (PDF) [file pone.0133753.s002.pdf]

Initial questionnaire to the public

The project "I want more and better cells!", funded by COMPETE, and developed by Center for Neuroscience and Cell Biology researchers, resulted in the production of outreach materials in the stem cells field.

This questionnaire will be used to study the impact of the materials in society, and to analyze if the objectives were fulfilled.

All the information is confidential and will be used for research purposes only.

Thank you for your participation.

Age: ____

Job: ____

Gender: ____

1. Mention 3 words or expressions that you link with stem cells.

2. What do you know about stem cells?

____

____

____

____

____

____

____

3. Answer “True” or “False” or “I don´t know” to the following statements

a) After born, the organism keeps the cellular renewal ability (the capability of cells to renewal).

☐ True ☐ False ☐ I don´t know

b) All stem cells are equal.

☐ True ☐ False ☐ I don´t know

c) All stem cells are located in the umbilical cord blood.

☐ True ☐ False ☐ I don´t know

d) All stem cells are located in the bone marrow.

☐ True ☐ False ☐ I don´t know

e) Induced pluripotent stem cells (IPSCs) could be an important tool to study neurodegenerative disorders such as Alzheimer and Parkinson diseases.

☐ True ☐ False ☐ I don´t know

f) We can use stem cells for therapy in cardiac diseases, such as a heart stroke.

☐ True ☐ False ☐ I don´t know

g) Stem cells could have an important role in drug discovery and allow the development of personalized medicines, i.e., drugs specifically designed for each patient, considering his particular case.

☐ True ☐ False ☐ I don´t know

4. Select the option(s) that you consider correct to complete the following statements / questions (select one or more options).

a) Physical and mental exercises lead to the formation of new neurons because in brain there are…

☐ Neural stem cells ☐ Hematopoietic stem cells ☐ Neurons ☐ Embryonic stem cells ☐ I don´t know

b) What is / are the special propriety (ies) of stem cells?

☐ Electric isolation ☐ Self-renewal ☐ Body protection ☐ Differentiation ability ☐ I don´t know

c) How much time (in average) is needed to produce an effective and safety drug?

☐ 1 year ☐ 5 years ☐ 10 years ☐ 30 years ☐ I don´t know

d) Stem cells can be…

☐ Pluripotent:	☐ Multipotent:	☐ Unipotent:	☐ I don´t know
Can give rise to all types of cells in the body (brain, blood, heart, etc.)	Can give rise to (specific cell types, according to the place where they are (different types of blood cells, different types of nervous system cells, etc.)	Can only give rise to one kind of cells	

Final questionnaire to the public

The project “I want more and better cells!”, funded by COMPETE, and developed by Center for Neuroscience and Cell Biology researchers, resulted in the production of outreach materials in the stem cells field.

This questionnaire will be used to study the impact of the materials in society, and to analyze if the objectives were fulfilled.

All the information is confidential and will be used for research purposes only.

Thank you for your participation.

Age: ____

Job: ____

Gender: ____

1. Mention 3 words or expressions that you link with stem cells.

2. What do you know about stem cells?

____

____

____

____

____

____

____

3. Answer “True” or “False” or “I don´t know” to the following statements

a) After born, the organism keeps the cellular renewal ability (the capability of cells to renewal).

☐ True ☐ False ☐ I don´t know

b) All stem cells are equal.

☐ True ☐ False ☐ I don´t know

c) All stem cells are located in the umbilical cord blood.

☐ True ☐ False ☐ I don´t know

d) All stem cells are located in the bone marrow.

☐ True ☐ False ☐ I don´t know

e) Induced pluripotent stem cells (IPSCs) could be an important tool to study neurodegenerative disorders such as Alzheimer and Parkinson diseases.

☐ True ☐ False ☐ I don´t know

f) We can use stem cells for therapy in cardiac diseases, such as a heart stroke.

☐ True ☐ False ☐ I don´t know

g) Stem cells could have an important role in drug discovery and allow the development of personalized medicines, i.e., drugs specifically designed for each patient, considering his particular case.

☐ True ☐ False ☐ I don´t know

4. Select the option(s) that you consider correct to complete the following statements / questions (select one or more options).

a) Physical and mental exercises lead to the formation of new neurons because in brain there are…

☐ Neural stem cells ☐ Hematopoietic stem cells ☐ Neurons ☐ Embryonic stem cells ☐ I don´t know

b) What is / are the special propriety (ies) of stem cells?

☐ Electric isolation ☐ Self-renewal ☐ Body protection ☐ Differentiation ability ☐ I don´t know

c) How much time (in average) is needed to produce an effective and safety drug?

☐ 1 year ☐ 5 years ☐ 10 years ☐ 30 years ☐ I don´t know

d) Stem cells can be…

☐ Pluripotent:	☐ Multipotent:	☐ Unipotent:	☐ I don´t know
Can give rise to all types of cells in the body (brain, blood, heart, etc.)	Can give rise to (specific cell types, according to the place where they are (different types of blood cells, different types of nervous system cells, etc.)	Can only give rise to one kind of cells	

5. Understanding of scientific topics (choose only one option)

a) The materials transmit the scientific message in a clear way.

☐ I totally disagree ☐ I disagree ☐ I don´t disagree/agree ☐ I agree ☐ I totally agree

b) The materials transmit the scientific message in a challenging way.

☐ I totally disagree ☐ I disagree ☐ I don´t disagree/agree ☐ I agree ☐ I totally agree

c) I learned new concepts and processes in the stem cell field.

☐ I totally disagree ☐ I disagree ☐ I don´t disagree/agree ☐ I agree ☐ I totally agree

d) The acquired knowledge allows me to do a more critical analysis of these issues.

☐ I totally disagree ☐ I disagree ☐ I don´t disagree/agree ☐ I agree ☐ I totally agree

e) Which material has contributed more to the acquisition of knowledge and understanding about this topic?

☐ Comics ☐ Newspaper illustrated chronicles ☐ Radio interviews ☐ Animated videos

6. **Engagement with science** (choose only one option)

a) The materials enhanced the curiosity about these topics.

☐ I totally disagree ☐ I disagree ☐ I don´t disagree/agree ☐ I agree ☐ I totally agree

b) The materials enhanced the interest in learning more and searching for new knowledge sources.

☐ I totally disagree ☐ I disagree ☐ I don´t disagree/agree ☐ I agree ☐ I totally agree

c) Which material has contributed more to the engagement in science?

☐ Comics ☐ Newspaper illustrated chronicles ☐ Radio interviews ☐ Animated videos

7. Attitude toward scientific issues (choose one option)

a) Did you already have any idea about this scientific topic?

☐ Yes ☐ No

If you chose **Yes**, answer questions **a1 and a2**.

If you chose **No**, answer the question **a3**.

a1. Your conception was:<br>☐ Very negative<br>☐ Negative<br>☐ Neither negative nor positive<br>☐ Positive<br>☐ Very positive<br><br>a2. Do you think that these materials helped you to clarify puzzled concepts?<br>☐ I totally disagree<br>☐ I disagree<br>☐ I don´t disagree/agree<br>☐ I Agree<br>☐ I totally agree	a3. At the moment, your conception about this topic is:<br>☐ Very negative<br>☐ Negative<br>☐ Neither negative nor positive<br>☐ Positive<br>☐ Very positive

b) Do you think that these materials can help you in the decision making process (personal, social and politic)?

☐ I totally disagree ☐ I disagree ☐ I don´t disagree/agree ☐ I agree ☐ I totally agree

c) Imagine that you would have to decide for the cryopreservation (or not) of your baby´s umbilical cord stem cells, or decide if you would accept a therapy using stem cells to treat a diseased relative. Do you think that these materials would influence your opinion or decision?

☐ I totally disagree ☐ I disagree ☐ I don´t disagree/agree ☐ I agree ☐ I totally agree

d) In your opinion, which level of importance does stem cell research have?

☐ Unimportant ☐ Not very important ☐ Relatively important ☐ Important ☐Very important

Thank you for you participation!
