# Supplementary material for: I Want More and Better Cells! – An Outreach Project about Stem Cells and Its Impact on the General Population
Source: PLoS One. 2015 Jul 29;10(7):e0133753. doi: 10.1371/journal.pone.0133753 (PMC4519251; doi:10.1371/journal.pone.0133753)
Supplement: S3 File — The table contains the rotated factor loadings, which represent how the items are weighted for each requested factor (F1, F2, F3), and also the correlation between the items and the factor. The mean (M) and standard deviation (SD) for each item are also shown. (PDF) [file pone.0133753.s003.pdf]

Rotated factor matrix obtained by an exploratory factor analysis with varimax rotation for 8 items. - The table contains the rotated factor loadings, which represent how the items are weighted for each requested factor (F1, F2, F3), and also the correlation between the items and the factor. The mean (*M*) and standard deviation (*SD*) for each item are also shown.

Items	F1	F2	F3	M	SD
Clarity [question 5.a)]*	**0.87**	0.18	0.12	4.15	0.66
Interest [question 5.b)]*	**0.85**	0.21	0.17	4.04	0.75
Critical thinking [question 5.d)]*	**0.48**	0.37	0.42	3.87	0.82
Learning of new concepts [question 5.c)]*	0.46	**0.61**	0.25	4.280	0.64
Curiosity [question 6.a)]*	0.13	**0.90**	0.35	3.95	0.68
Wish to learn more [question 6.b)]*	0.26	**0.64**	0.40	3.78	0.70
Attitude (personal/socio-political decisions) [question 7.b)]*	0.12	0.41	**0.73**	3.96	0.63
Attitude (personal decisions) [question 7.c)]*	0.19	0.24	**0.90**	4.04	0.70

* Questions from the final questionnaire to the public (S2_File)
