# Supplementary material for: I Want More and Better Cells! – An Outreach Project about Stem Cells and Its Impact on the General Population
Source: PLoS One. 2015 Jul 29;10(7):e0133753. doi: 10.1371/journal.pone.0133753 (PMC4519251; doi:10.1371/journal.pone.0133753)
Supplement: S4 File — (PDF) [file pone.0133753.s004.pdf]

Pearson´s Correlations among all variables analyzed for the high-school students, university students, active population, and retired population.

High-school students				
	Understanding	Engagement	Attitude	Knowledge score
Understanding	1			
Engagement	0.554**	1		
Attitude	0.531**	0.411**	1	
Knowledge score	0.304*	0.241	0.203	1

University students				
	Understanding	Engagement	Attitude	Knowledge score
Understanding	1			
Engagement	0.402**	1		
Attitude	0.245	0.309*	1	
Knowledge score	0.099	0.189	0.045	1

Active population				
	Understanding	Engagement	Attitude	Knowledge score
Understanding	1			
Engagement	0.461**	1		
Attitude	0.323*	0.537**	1	
Knowledge score	0.095	-0.163	0.121	1

Retired population				
	Understanding	Engagement	Attitude	Knowledge score
Understanding	1			
Engagement	0.787**	1		
Attitude	0.589**	0.669**	1	
Knowledge score	0.308*	0.093	0.195	1

** Correlations are significant at the 0.01 level (2-tailed)

* Correlation is significant at the 0.05 level (2-tailed)
